# Supplementary material for: Analgesic effect of diluted nitrous oxide in rehabilitation training of patients with burn scar contracture: Study protocol for a randomized controlled trial
Source: PLoS One. 2025 Sep 25;20(9):e0333378. doi: 10.1371/journal.pone.0333378 (PMC12463218; doi:10.1371/journal.pone.0333378)
Supplement: S2 File — (DOCX) [file pone.0333378.s002.docx]

**研究方案**

**项目名称：**稀释氧化亚氮在烧伤术后瘢痕挛缩患者康复训练中镇痛效果的干预研究

**项目来源：**山东第二医科大学附属医院科技发展项目

**开展时间：**2023.12～2026.12

**开展地点：**中国人民解放军联勤保障部队第九六〇医院

**主要研究者：**唐鲁、王飞、宗晶、曲桂玉、姜玉晓、孙晨曦、邱雪玲、王伟峰、王自阳、孙凡、蒋晓晨、李翠

**联系方式：**15064003655

1. **概述**

康复训练是烧伤患者术后必不可少的治疗环节。患者最终康复效果取决于能否进行早期康复训练, 但患者常因瘢痕挛缩出现剧烈疼痛, 很难保证锻炼的进行, 严重影响患者日常生活活动能力和生活质量。本项目旨在探讨稀释氧化亚氮镇痛技术用于烧伤术后康复训练的安全性和有效性，为烧伤患者康复训练时期减轻或免除疼痛提供实证依据。

**二、研究对象**

1.样本量的计算：根据样本量计算，经咨询专家后，将样本量确定为120人次，干预组60人次，对照组60人次。

2.纳入标准：经过伦理审批后，根据预试验结果、按照研究设置纳入标准：年龄＞18岁；疼痛评分VAS≥ 4的烧伤术后康复训练的患者；患者外伤不影响使用多功能心电监护仪或脉搏氧监测器；自愿参加并签署知情同意书；患者神志清楚，能够正确理解和使用视觉模拟疼痛评分工具表达疼痛程度。

3.排除标准：中耳炎、鼻窦炎、肠梗阻；急性上呼吸道感染或其他原因导致的呼吸不畅；VitB12缺乏症;美国麻醉医师协会健康状况分级（ASA）在III级以上的病人;药物滥用、酗酒、严重精神疾病或异常;幽闭恐惧症或鼻罩不耐受;慢性阻塞性肺疾病、气胸、肺大泡;亚甲基四氢叶酸还原酶缺乏症;重度睡眠呼吸暂停综合征的病人

**三、研究内容和方法**

**试验设计:**

采用质性研究和病例回顾研究相结合的方法，了解我国烧伤术后瘢痕挛缩康复训练中疼痛时的控制现状。设计随机双盲对照研究设计，探索稀释氧化亚氮对烧伤术后康复训练的镇痛作用。总结经验，编写该技术的规范化操作规程，培训人员，将其成熟技术在多个基层医疗机构推广使用。

**研究方法:**

1.质性研究：，对某三级甲等医院工作三年以上的临床一线医务人员、烧伤术后出现瘢痕挛缩接受康复训练的患者采用立意抽样，以面对面、半结构式、深度访谈的方式收集资料。

2.量性研究：采用随机双盲对照试验，，通过观察检测生理指标（疼痛评分（VAS）、焦虑评分（VAS）、镇静程度（Ramsay）、瘢痕评分、一般功能评分（ADL）、医生及患者满意度、血压（BP）、心率（HR）、血氧饱和度（SPO2），探讨稀释氧化亚氮对烧伤术后瘢痕挛缩患者康复训练的镇痛作用及其机制。

**四、预期研究成果**

1. 编撰该适宜技术的护理操作规程。
2. 培训项目工作人员。
3. 培训一批临床疼痛专科医护人员。
4. 发表SCI论文1-2篇，申请成果鉴定。
5. 培养研究生2名。

**五、本研究可能出现的不良事件**

主要的副作用包括呼吸系统（如氧饱和度降低、气道阻塞、呼吸暂停）、心血管系统（如心脏病）和过度呼吸（即恢复时间超过5分钟）。轻微的副作用包括恶心呕吐、欣快、头晕和感觉迟钝。长时间反复吸入可能导致永久性神经功能障碍的不可逆性周围性脊髓神经病。

**不良事件的风险评估**

1、积极采取补救或抢救措施并妥善保管有关记录、标本、化验成效及相关的药品、器械。

2、当事人立即报告值班医师、护士长、主任。

3、科室填写护理不良事件报告单，记录不良事件经由、原因、后果及本人对不良事件的认识和建议。

4、对事件进行调查，组织科内讨论，分析管理制度、工作流程及层级管理方面存在的问题，确定事件的原因，提出改进措施，并将报告表上交护理部及医务部，定期跟踪改进措施的落实。

**不良事件的应对措施**

立即停止吸入氧化亚氮，给予患者100%纯氧吸入，3-5分钟即可缓解。

**知情同意书**

**尊敬的女士/先生：您好！**

我们将邀请您参加“稀释氧化亚氮在烧伤术后瘢痕挛缩患者康复训练中镇痛效果的干预研究”。如果您同意加入此项研究，请您仔细阅读下面的内容，充分考虑后再决定是否参加该研究。**本知情同意书已通过中国人民解放军联勤保障部队第九六〇医院医学伦理委员会审查。**如有任何疑问请您随时向负责该项研究的研究人员提出。

**一、研究项目简介**

混合气体速效镇痛适宜技术的原理是利用速效镇痛气体，即[一氧化二氮](http://zh.wikipedia.org/wiki/%E4%B8%80%E6%B0%A7%E5%8C%96%E4%BA%8C%E6%B0%AE)和氧气预先混合的气体，以[需求阀](http://zh.wikipedia.org/w/index.php?title=%E9%9C%80%E6%B1%82%E9%96%A5&action=edit&redlink=1)形式通过患者自行供气而实现镇痛及镇静作用的。国际上该混合气体以Kalinox, Medimix, Antasol, Oxynox等商品名被广泛应用于临床和院前急救镇痛，如胸部挤压伤、心脏手术后、烧伤换药、乙状结肠镜检、无痛分娩、无痛人工流产、牙科镇痛、肝组织活检、前列腺组织活检、癌性疼痛、减轻急诊患儿的疼痛和焦虑、急救车接诊时对冠心病患者以及终末期患者的顽固性疼痛的镇痛处理等方面。自[19世纪](http://zh.wikipedia.org/wiki/1800%E5%B9%B4%E4%BB%A3)末英国将该技术首次应用于无痛分娩开始，吸入镇痛技术在欧洲广泛普及。英国将该混合气体以ENTONOX名商品化并用于临床镇痛，此后在法国、瑞典、加拿大、美国、澳大利亚等73个国家得到了广泛应用。国内自2005年起将其用于妇科、牙科和腔镜检查、烧伤换药的镇痛镇静。

本课题研究速效镇痛适宜技术在洁牙中的应用,建立健全一种安全有效、操作简便、价格低廉、清醒无痛的镇痛、镇静方法，为洁牙操作疼痛的患者镇痛、镇静提供参考。

1. **受试者准入与排除标准**

患者的录入标准：经过伦理审批后，根据预试验结果、按照研究设置纳入标准：年龄＞18岁；疼痛评分VAS≥ 4的烧伤术后康复训练的患者；患者外伤不影响使用多功能心电监护仪或脉搏氧监测器；自愿参加并签署知情同意书；患者神志清楚，能够正确理解和使用视觉模拟疼痛评分工具表达疼痛程度。

排除标准如下: 中耳炎、鼻窦炎、肠梗阻；急性上呼吸道感染或其他原因导致的呼吸不畅；VitB12缺乏症;美国麻醉医师协会健康状况分级（ASA）在III级以上的病人;药物滥用、酗酒、严重精神疾病或异常;幽闭恐惧症或鼻罩不耐受;慢性阻塞性肺疾病、气胸、肺大泡;亚甲基四氢叶酸还原酶缺乏症;重度睡眠呼吸暂停综合征的病人

获得所有参与者的知情同意。取得机构审查委员会许可。

**三、研究可能的受益**

按照本研究的实施方案，研究人员会对您实施稀释氧化亚氮速效镇痛适宜技术的规范化操作，这种方法有利于减轻疼痛的折磨和伤害，促进快速康复。

**四、参加本项目的风险及补偿措施**

本项研究不会对您的身体、心理及社会关系等造成任何伤害，更不会对您原有疾病的诊断、化验、检查、用药及其他治疗产生任何不便及负面影响；您也不会因参加本研究而获得任何酬劳。整个研究过程接受中国人民解放军联勤保障部队第九六〇医院医学伦理委员会的监督，研究过程中如遇到任何疑问可随时向研究人员咨询。

您在参加研究期间不会受到因为本次研究造成的任何损害。即使您已经签署这份知情同意书，您仍然保留您所有的合法权利。

**五、研究资料的保密性**

个体生物样本涉及私人疾病信息、生命信息、基因信息等。如果您决定参加本项研究，您参加试验及在试验中的个人资料均属保密。您的医疗记录（研究病历/CRF、化验单等）将完整保存在中国人民解放军联勤保障部队第九六〇医院，研究项目组成员、研究主管部门将被允许查阅您的医疗记录。为确保研究按规定进行，必要时，政府管理部门或伦理审查委员会的成员按规定可以被允许在研究单位内查阅您的个人资料。任何有关本研究项目的公开报告将不会披露您的个人身份。我们将在法律允许的范围内，尽一切努力保护您个人医疗资料和疾病信息、生命信息、基因信息的隐私。关于您的个人和医疗信息将对外保密，且被保管在安全可靠的地方。在任何时候，您可以随时查阅您的个人信息（比如您的姓名和地址），如有需要可以修改这些信息。本研究小组将严格遵循赫尔辛基宣言和世界卫生组织与国际医学科学组织理事会共同指定的《涉及人的生物医学研究国际伦理准则》的原则。

当您签署了这份知情同意书，即代表您同意您的个人和医疗信息被用于上述所描述的场合，您的档案将保存在中国人民解放军联勤保障部队第九六〇医院病案室档案柜中，仅供研究人员查阅。此项研究结果发表时，我们不会公开您的个人医疗资料信息。

**六、您的权力**

您参与本次科研项目是完全自愿的，您可以拒绝参加研究，或在研究过程中的任何时候选择退出研究，不需任何理由，该决定不会影响您以后的诊断及治疗，不会影响您和医务人员的关系。研究期间如有疑问或需要咨询相关问题时,您可以随时与您的主管医师联系。

参加本研究期间您可以随时选择：1. 不参加本研究，继续您的常规治疗；2.参加别的研究；3.不接受任何治疗。

**志愿受试者声明：**

我已详细阅读了“稀释氧化亚氮在烧伤术后瘢痕挛缩患者康复训练中镇痛效果的干预研究”的知情同意书，研究医师已向我作了详尽的研究方案说明，我完全了解参加本次研究的目的、性质、方法及我的权益和风险，得知我的个人资料是保密的，隐私权也得到保护。

我自愿参加本次研究，并同意按照研究方法和知情同意书的内容配合医生操作，认真完成本次研究。本知情同意书共4页，我将得到签名后的知情同意书复印件。

受试者签字： 日期： 联系电话：

我已向该受试者充分解释和说明了本临床试验的目的、操作过程以及受试者参加该试验可能存在的风险和潜在的利益，并满意地回答了受试者的所有有关问题。

研究者签字： 日期： 联系电话：

Study Protocol

Project title: Intervention study on analgesic effect of diluted nitrous oxide in rehabilitation training of patients with scar contracture after burn surgery

Project source: Science and Technology Development Project of Affiliated Hospital of Shandong Second Medical University

Development time: 2023.12 ~ 2026.12

Location: The 960th Hospital of the Joint Logistics Support Force of the Chinese People's Liberation Army

Principal Investigators: Tang Lu, Wang Fei, Zong Jing, Qu Guiyu, Jiang Yuxiao, Sun Chenxi, Qiu Xueling, Wang Weifeng, Wang Ziyang, Sun Fan, Jiang Xiaochen, Li Cui

Contact information: 15064003655

I. Overview

Rehabilitation training is an essential treatment link for burn patients after surgery. The final rehabilitation effect of patients depends on whether early rehabilitation training can be carried out, but patients often suffer from severe pain due to scar contracture, which makes it difficult to ensure the exercise, which seriously affects patients' activities of daily living and quality of life. The purpose of this project is to explore the safety and effectiveness of diluted nitrous oxide analgesia in rehabilitation training after burn surgery, and to provide empirical evidence for reducing or avoiding pain in burn patients during rehabilitation training.

2. Research objects

1). Calculation of sample size: According to the sample size calculation, after consulting experts, the sample size was determined to be 120 person-times, 60 person-times in the intervention group and 60 person-times in the control group.

2). Inclusion criteria: After ethical approval, according to the pre-trial results and the inclusion criteria according to the research setting: age > 18 years old; Patients with post-burn rehabilitation training with pain score VAS 4; Patient trauma does not affect the use of multifunctional ECG monitors or pulse oxygen monitors; Voluntarily participate and sign the informed consent form; The patient was conscious and able to correctly understand and use the visual analog pain score tool to express pain degree.

3). Exclusion criteria: otitis media, sinusitis, intestinal obstruction; Acute upper respiratory tract infection or poor breathing caused by other reasons; VitB12 deficiency; Patients with American Society of Anesthesiologists Health Status Scale (ASA) level III or above; Drug abuse, alcohol abuse, serious mental illness or abnormalities; Claustrophobia or nasal mask intolerance; Chronic obstructive pulmonary disease, pneumothorax, bullae; Methylenetetrahydrofolate reductase deficiency; Patients with severe sleep apnea syndrome

3. Research content and methods

Experimental design:

To understand the current situation of pain control in rehabilitation training of scar contracture after burn surgery in China by combining qualitative study with case review study. Design a randomized double-blind controlled study design to explore the analgesic effect of diluted nitrous oxide on rehabilitation training after burn surgery. Summarize experience, compile standardized operating procedures of this technology, train personnel, and popularize and use its mature technology in many primary medical institutions.

Research Methods:

1). Qualitative research: The clinical frontline medical staff who have worked in a tertiary hospital for more than three years and the patients who have scar contracture after burn surgery and receive rehabilitation training were collected by intentional sampling, and the data were collected by face-to-face, semi-structured and in-depth interview.

2). Quantitative study: A randomized double-blind controlled trial was used to observe and detect physiological indexes (pain score (VAS), anxiety score (VAS), sedation degree (Ramsay), scar score, general function score (ADL), doctor and patient satisfaction, blood pressure (BP), heart rate (HR), blood oxygen saturation (SPO2), to explore the analgesic effect and mechanism of diluted nitrous oxide on rehabilitation training of patients with scar contracture after burn operation.

4. Expected research results

1). Compile the nursing operating procedures of this appropriate technology.

2). Train project staff.

3). Train a group of clinical pain specialists.

4). Publish 1-2 SCI papers and apply for achievement appraisal.

5). Train 2 graduate students.

5. Possible adverse events in this study

Major side effects include respiratory system (e.g., decreased oxygen saturation, airway obstruction, apnea), cardiovascular system (e.g., heart disease), and hyperbreathing (i.e., recovery time longer than 5 minutes). Minor side effects include nausea and vomiting, euphoria, dizziness, and dullness. Irreversible peripheral myeloneuropathy in which repeated inhalation for a long time may cause permanent neurological dysfunction.

Risk assessment of adverse events

1. Actively take remedial or rescue measures and properly keep relevant records, specimens, laboratory results and related drugs and equipment.

2. The parties concerned immediately report to the doctor, head nurse and director on duty.

3. The department fills in the nursing adverse event report form to record the course, cause, consequence of the adverse event, as well as my understanding and suggestions on the adverse event.

4. Investigate the incident, organize the discussion within the department, analyze the problems existing in the management system, work flow and hierarchical management, determine the cause of the incident, propose improvement measures, and submit the report form to the nursing department and medical department to regularly track the implementation of improvement measures.

Countermeasures for adverse events

Stop the inhalation of nitrous oxide immediately and give the patient 100% pure oxygen inhalation, which can be relieved in 3-5 minutes.

Informed consent form

Dear Madam/Sir: Hello!

You will be invited to participate in the "Intervention Study on the Analgesic Effect of Diluted Nitrous Oxide in Rehabilitation Training of Patients with Scar Contracture after Burn Operation". If you agree to participate in this study, please read the following details carefully and consider them thoroughly before deciding whether to participate in this study. This informed consent form has been reviewed by the Medical Ethics Committee of the No.960 Hospital of the Joint Logistics Support Force of the Chinese People's Liberation Army. Please feel free to send any questions to the investigator in charge of the study.

1. Introduction to research projects

The principle of the appropriate technology of mixed gas quick-acting analgesia is to use quick-acting analgesic gas, that is, the pre-mixed gas of nitrous oxide and oxygen, to achieve analgesia and sedation by supplying gas by the patient himself in the form of a demand valve. Internationally, the mixed gas is widely used in clinical and pre-hospital emergency analgesia under the trade names of Kalinox, Medimix, Antasol, Oxynox, etc., such as chest crush injury, cardiac surgery, burn dressing change, sigmoidoscopy, painless delivery, painless artificial abortion, dental analgesia, liver tissue biopsy, prostate tissue biopsy, cancer pain, pain and anxiety relief of emergency children, analgesic treatment of intractable pain of patients with coronary heart disease and end-stage patients during ambulance admission, etc. Since the technology was first applied to painless delivery in Britain at the end of 19th century, inhalation analgesia technology has been widely used in Europe. The United Kingdom commercialized the mixed gas under the name ENTONOX and used it for clinical analgesia. Since then, it has been widely used in 73 countries including France, Sweden, Canada, the United States, and Australia. Since 2005, it has been used for analgesia and sedation in gynecology, dentistry, endoscopy and burn dressing change in China.

This topic studies the application of suitable quick-acting analgesia techniques in tooth scaling, and establishes and improves a safe, effective, easy to operate, low-cost, awake and painless analgesia and sedation method, so as to provide reference for analgesia and sedation of patients with pain in tooth scaling operation.

2. Subject admission and exclusion criteria

Patient entry criteria: after ethical approval, according to the pre-trial results and the inclusion criteria according to the study setting: age > 18 years old; Patients with post-burn rehabilitation training with pain score VAS 4; Patient trauma does not affect the use of multifunctional ECG monitors or pulse oxygen monitors; Voluntarily participate and sign the informed consent form; The patient was conscious and able to correctly understand and use the visual analog pain score tool to express pain degree.

The exclusion criteria were as follows: otitis media, sinusitis, intestinal obstruction; Acute upper respiratory tract infection or poor breathing caused by other reasons; VitB12 deficiency; Patients with American Society of Anesthesiologists Health Status Scale (ASA) level III or above; Drug abuse, alcohol abuse, serious mental illness or abnormalities; Claustrophobia or nasal mask intolerance; Chronic obstructive pulmonary disease, pneumothorax, bullae; Methylenetetrahydrofolate reductase deficiency; Patients with severe sleep apnea syndrome

Informed consent was obtained from all participants. Obtain permission from the Institutional Review Board.

3. Possible benefits of research

According to the implementation protocol of this study, the researchers will perform standardized operation of appropriate techniques for rapid-acting analgesia with diluted nitrous oxide. This method is beneficial to alleviate pain torture and injury and promote rapid recovery.

4. Risks and compensation measures for participating in this project

This study will not cause any harm to your physical, psychological and social relationships, nor will it cause any inconvenience or negative impact on the diagnosis, laboratory tests, examinations, medication and other treatments of your pre-existing diseases; You will also not receive any remuneration for participating in this study. The whole research process is under the supervision of the Medical Ethics Committee of the No.960 Hospital of the Joint Logistics Support Force of the Chinese People's Liberation Army. If you encounter any questions during the research process, you can consult the researchers at any time.

You will not suffer any harm from this study during your participation in the study. Even if you have signed this informed consent form, you still retain all your legal rights.

5. Confidentiality of research data

Individual biological samples involve private disease information, life information, genetic information, etc. If you decide to participate in this study, your personal data about participation in the trial and during the trial will be confidential. Your medical records (research medical records/CRFs, laboratory test sheets, etc.) will be kept completely in the 960th Hospital of the Joint Logistics Support Force of the Chinese People's Liberation Army, and members of the research project team and research authorities will be allowed to consult your medical records. In order to ensure that the research is carried out according to regulations, when necessary, members of government management departments or ethics review committees may be allowed to access your personal data within the research unit. Any public reports about this research project will not disclose your personal identity. We will make every effort to protect the privacy of your personal medical data, disease information, vital information and genetic information within the scope permitted by law. Personal and medical information about you will be kept confidential and kept in a safe and secure place. At any time, you can consult your personal information (such as your name and address) at any time and modify this information if necessary. This research team will strictly follow the principles of the Helsinki Declaration and the International Ethical Guidelines for Biomedical Research Involving Humans jointly designated by the World Health Organization and the International Council of Medical Scientific Organizations.

When you sign this informed consent form, you agree that your personal and medical information will be used for the occasions described above. Your file will be stored in the medical record room of the No. 960 Hospital of the Joint Logistics Support Force of the Chinese People's Liberation Army. In the file cabinet, it is only available to researchers. When the results of this study are published, we will not disclose your personal medical data.

6. Your rights

Your participation in this scientific research project is completely voluntary. You can refuse to participate in the research, or choose to withdraw from the research at any time during the research process without any reason. This decision will not affect your future diagnosis and treatment, and will not affect your relationship with medical staff. If you have questions or need to consult related questions during the study, you can always contact your supervising physician.

You can choose at any time during this study: 1. Do not participate in this study and continue your usual treatment; 2. Participate in other studies; 3. Do not receive any treatment.

Volunteer Subject Statement:

I have read the informed consent form of "Intervention Study on Analgesic Effect of Diluted Nitrous Oxide in Rehabilitation Training of Patients with Scar Contracture after Burn Surgery" in detail, and the research doctor has given me a detailed explanation of the research protocol. I fully understand the purpose, nature, method, my rights and risks of participating in this study, and know that my personal data are confidential and my privacy rights are protected.

I volunteered to participate in this study, and agreed to cooperate with the doctor in accordance with the study methods and the contents of the informed consent form, and carefully complete this study. This informed consent form consists of 4 pages, and I will be given a signed copy of the informed consent form.

Subject Signature: Date: Tel:

I have fully explained and explained to the subject the purpose of this clinical trial, the operation process, the possible risks and potential benefits of the subject's participation in this trial, and satisfactorily answered all relevant questions of the subject.

Investigator Signature: Date: Tel:
