# Supplementary material for: Analgesic effect of diluted nitrous oxide in rehabilitation training of patients with burn scar contracture: Study protocol for a randomized controlled trial
Source: PLoS One. 2025 Sep 25;20(9):e0333378. doi: 10.1371/journal.pone.0333378 (PMC12463218; doi:10.1371/journal.pone.0333378)
Supplement: Attachment — Submitted filename: Response to Reviewers.docx [file pone.0333378.s004.docx]

**To editor:**

Thank you very much for helping us point out the problem, here is my response item by item.If you still have any questions, please feel free to contact me. I wish you the best!

1 The manuscript has been revised to meet the style requirements of PLOS ONE

2 I have removed the ethical statement from the manuscript except the method section.

3 I have added caption at the reference figure

4 Shandong Second Medical University The previous name of the university was Weifang Medical University. After this project was approved, the school was renamed Shandong Second Medical University. I have changed the funding information in the manuscript to Weifang Medical University. If you still need to modify Please let me know.

**Comments from other editors:**

1 In the method-Participants, "All patients with pain caused by rehabilitation training after burn surgery will be invited to participate in this study." We then described the inclusion criteria: "Worst pain reported during rehabilitation exercise ≥ 4 (0-10 cm on the visual analog scale (VAS))." Different patients have differences in their condition and pain degree. The main purpose of this study is to reduce acute pain during rehabilitation training in burn patients, so we took pain degree as an inclusion criterion. This inclusion criterion explains this sentence.

2 The rehabilitation of burn patients needs professional rehabilitation trainers to complete it. The subjects of this study are hospitalized patients.

3 Visual analog scale has the advantages of accuracy, simplicity and high sensitivity, and is widely used in clinical and scientific research work. Another advantage of VAS is that its value changes continuously and can be used for parameter verification. The use of digital rating scale requires patients to have certain abstract scale comprehension ability and word reading comprehension ability. Therefore, in order to avoid subsequent special situations, we chose to use visual analog scales.

4 conventional treatment approach I have added to the interventions section of the manuscript.

**Reviewer 1:**

The question you asked is very important, thank you so much! We have made changes

1. We have described the calculation of sample size in more detail in the Sample size estimation section of the article. The calculation results show that when the sample size of the group is 18 (9 cases in each group), it can meet the requirements of the parameters in this paper. However, as requested by the State Food and Drug Administration, to find out if the gas had more side effects and to increase the credibility of the study, we expanded the sample size to 120 (60 cases per group)

2. I have added details about rehabilitation exercises to the intervention section. In terms of outcome indicators, some editors also raised this issue. We used the visual analog scale (VAS) to assess the degree of pain because it has the advantages of accuracy, simplicity and high sensitivity. In fact, there are more accurate measurement tools such as biomarkers, but because we have many measurement nodes, multiple blood sampling in a rehabilitation training may arouse patient resentment, and the inflammatory response of burn patients may affect its accuracy. Therefore, we currently believe that VAS is an economical, simple and accurate measurement tool suitable for this test. We will also monitor the changes of patients' blood pressure and heart rate to assist the test.

3. We do ignore this point. The problems actually faced by nitrous oxide applications have been described in the discussion section.

**Reviewer 2:**

The problems you pointed out can make up for our great shortcomings. Thank you very much for your careful review. We have made corrections.

1. This has been corrected in the article. The calculation of sample size is described in more detail

2. Details about rehabilitation exercises have been added to the intervention section.

3. T1 is a time point at which gas inhalation is started, and T4 is a time point at which gas inhalation is stopped. The onset time of nitrous oxide is mentioned in our discussion section from 30 seconds to two minutes, and the settings of T2(two minutes after inhalation) and T3 (five minutes after inhalation) are determined according to this pharmacological property. There is a real problem with the description of T5, which we have revised to four weeks after intervention. In Fig.1, it is clear at which time point each outcome measure was evaluated.

4. We did ignore this point, and we have added a relevant description in the limitations section.

5. We added this to the Data Availability section.
