# Supplementary material for: Analgesic effect of diluted nitrous oxide in rehabilitation training of patients with burn scar contracture: Study protocol for a randomized controlled trial
Source: PLoS One. 2025 Sep 25;20(9):e0333378. doi: 10.1371/journal.pone.0333378 (PMC12463218; doi:10.1371/journal.pone.0333378)
Supplement: Attachment — Submitted filename: Response_to_Reviewers_auresp_2.docx [file pone.0333378.s005.docx]

**To editor:**

We don't seem to find that you have raised a new question. Thank you very much for your help! Please feel free to contact me if you still have questions. I wish you all the best!

**Reviewer 1:**

Thank you so much for your recognition and help with our manuscript!

**Reviewer 2:**

What you said is really important, and we have corrected it in the text. Thank you so much for your questions!
