# Supplementary material for: Analgesic effect of diluted nitrous oxide in rehabilitation training of patients with burn scar contracture: Study protocol for a randomized controlled trial
Source: PLoS One. 2025 Sep 25;20(9):e0333378. doi: 10.1371/journal.pone.0333378 (PMC12463218; doi:10.1371/journal.pone.0333378)
Supplement: Attachment — Submitted filename: Response_to_Reviewers_auresp_3.docx [file pone.0333378.s006.docx]

**To editor:**

We don't seem to find that you have raised a new question. Thank you very much for your help! Please feel free to contact me if you still have questions. I wish you all the best!

**Reviewer 3:**

Thank you very much for your careful review, the questions you have raised are very important.

1. In the description of the measurement time point, changing "inhalation" to "intervention" is indeed clearer and more consistent, and we have modified it in the text.

2. There are indeed inconsistencies in the description of pain in the sample size estimation, which we have revised to the degree of pain reduction.

3. A clear description of the intervention time points we added to the sample size estimation section.

4. We have described the calculation of sample size in more detail in the Sample size estimation section of the article. The calculation results show that when the sample size of the group is 18 (9 cases in each group), it can meet the requirements of the parameters in this paper. This gas reported a low incidence of side effects and adverse reactions, as requested by the State Food and Drug Administration, to find out if the gas had more side effects, We expanded the sample size.A larger sample size can add credibility to the study, but the 120 sample size does present some difficulties during the collection process. Therefore, following your suggestion, we finally revised the sample size to 80 cases (40 cases per group).We expect 10 eligible patients to join the study each month.

5.Due to the purchase of gas and equipment, we delayed the recruitment time, and the article was not revised in time. Corrections have been made now.

6.The T0 and T3 time point data for the preliminary experiment were as follows (A: intervention group B: placebo group).This is only used to calculate sample size.

| VAS | 1 | 2 | 3 | 4 | 5 | 6 | 7 | 8 | 9 | 10 |
| --- | --- | --- | --- | --- | --- | --- | --- | --- | --- | --- |
| A(T0) | 8 | 9 | 8 | 6 | 7 | 8 | 5 | 6 | 8 | 6 |
| A(T3) | 6 | 6 | 5 | 4 | 5.5 | 5 | 4.5 | 5 | 4 | 3 |
| Δ | -2 | -3 | -3 | -2 | -1.5 | -3 | -0.5 | -1 | -4 | -3 |
| B(T0) | 8 | 7 | 6 | 7 | 5.5 | 7 | 6 | 6 | 7 | 7 |
| B(T3) | 6 | 6 | 5 | 6 | 5 | 6.5 | 5 | 5 | 5.5 | 6 |
| Δ | -2 | -1 | -1 | -1 | -0.5 | -0.5 | -1 | -1 | -1.5 | -1 |

It has been proposed in this paper that the sample size is calculated using PASS 15 software.

σ = √[(σ₁² + σ₂²)/2] d = (μ1 - μ2) / σ

Alternative Hypothesis= Two-Sided

Power= 0.9

Alpha= 0.05

Any help is greatly appreciated! I wish you all the best!
