# Supplementary material for: Analgesic effect of diluted nitrous oxide in rehabilitation training of patients with burn scar contracture: Study protocol for a randomized controlled trial
Source: PLoS One. 2025 Sep 25;20(9):e0333378. doi: 10.1371/journal.pone.0333378 (PMC12463218; doi:10.1371/journal.pone.0333378)
Supplement: Attachment — Submitted filename: Response_to_Reviewers_auresp_4.docx [file pone.0333378.s007.docx]

**To editor:**

We don't seem to find that you have raised a new question. Thank you very much for your help! Please feel free to contact me if you still have questions. I wish you all the best!

**Reviewer 3:**

Thank you very much for your careful review!We have made changes in the abstract.

I wish you all the best!
